# Integration of religious beliefs and faith-based resources in domestic violence services to migrant and ethnic minority communities: A scoping review

**DOI:** 10.1177/26330024241246810

**Published:** 2024-04-29

**Authors:** Romina Istratii, Parveen Ali, Gene Feder

**Affiliations:** SOAS University of London, UK; 7315 The University of Sheffield, and Doncaster and Bassetlaw Teaching Hospitals, UK; 152331 University of Bristol, Bristol Medical School, UK

**Keywords:** cultural competence, domestic abuse, domestic violence, integration, religious beliefs, religious mediators, service providers

## Abstract

Domestic violence and abuse (DVA) is a problem that cuts across borders and communities. There is an urgency for domestic violence service providers in multicultural societies like the United Kingdom (UK) to adapt to the diverse backgrounds of victims, survivors and perpetrators to design and provide appropriate support services and interventions. Religious beliefs are an integral part of many people’s lives and identities, while religious mediators often serve as a first point of reference for victimized parties to turn to, with both positive and negative impacts. It is currently unclear to what extent religious sensibilities are being addressed in DVA services, or how best to engage with religious beliefs and faith-based resources effectively. Recognizing this, we conducted a scoping review to identify existing approaches and practices for integrating religious beliefs and faith-based resources in domestic violence services. The review that had an international scope was conducted in English and included 30 publications. The synthesis of the evidence pointed to numerous approaches and efforts in integrating religious beliefs and faith-based resources in DVA services, differences and tensions in generalist and community-based responses, and the need for various measures in DVA services to cater to multicultural populations.

## Introduction

Domestic violence and abuse (DVA) is a problem that no community is immune to and one that cuts across borders. DVA refers to violence or abuse that occurs in a domestic setting, with intimate partner violence (IPV), which refers to abuse between current or former intimate partners, being the most prevalent form of DVA.^1^ There is no conclusive evidence that DVA is more prevalent in migrant and ethnic minority communities, including those that identify as religious, but such communities can be more vulnerable to DVA due to migration-related risks and stresses, in combination with colonial legacies, racism and other structural factors that can contribute to the problem and its continuation (Chantler et al. 2009; Du Monte and Forte, 2012; Holtmann 2016; Holtmann and Rickards, 2018; Istratii, 2023). Additionally, DVA victims and survivors^2^ may not access DVA services available to them due to a lack of cultural awareness and competence among service providers, victims’ and survivors’ fear of statutory agencies as a result of society of origin or in-transit experiences with state actors, survivors’ choice to rely on community-based resources to resolve the issue, or as a direct or indirect result of their religious beliefs (Holtmann and Rickards, 2018; Al-Natour et al., 2019; Pan et al., 2006; Khelaifat, 2019). Members of religious communities, including in migrant and emergency contexts, may instead turn to their faith as support, such as seeking mediation from religious leaders or relying on religious practices to cope, such as praying for the abuse to stop (Holtmann and Rickards, 2018; Al-Natour et al., 2019).

A review of the international evidence, combining religious studies, international development, public health and social work, evidences that religious beliefs can act both as a contributor to DVA and a deterrent to help-seeking and as a resource supporting survivors to cope and protection for people in their relationships (Istratii, 2020; Istratii and Ali, 2023; Le Roux and Pertek, 2023). It is also well-established that clergy and religious mediators, more broadly, are often the first to hear disclosures of DVA from survivors but often provide harmful advice or are unsupportive (Istratii, 2020; Johnson, 2015). A previous literature review that explored the relationship between religious beliefs and IPV and effective faith-informed community-based responses internationally, including DVA services to ethnic minority communities in industrialized societies and Gender-Based Violence (GBV) programs delivered with faith communities in low- and middle-income societies, pointed to an increasingly mainstreamed understanding that culturally appropriate, sensitive or competent services needed to engage with the religious beliefs of the communities involved in IPV programs and to leverage on the influence of religious teachers and clerics to respond effectively (Istratii and Ali, 2023).

Despite extensive evidence available on religion and IPV/GBV, the ways in which religious beliefs and faith-based resources have been integrated in the DVA sectors of societies where religious communities are minority or migrant populations are under-researched. The evidence on organized DVA services that effectively engage with the religious beliefs and experiences of survivors and perpetrators does not appear to have been systematically synthesized; to the authors’ knowledge, an effort is made by Walizadeh (2022) in relation to perpetrator treatment programs. In contrast to academic research, grey and organizational literature emanating from DVA service providers and platforms working to respond to DVA or Violence Against Women and Girls (VAWG) in the UK and other societies with diverse migrant and ethnic minority communities, such as the Unites States, Canada and Australia, features a significant gamut of manuals, toolkits and educational materials circulating online that seek to improve ‘religious literacy’ among service providers and to integrate faith actors in victim support systems, although these often lack theoretical depth and empirical evidence to inform academic research.

To-date, it is unclear to what extent religious sensibilities are being addressed in the DVA sectors of these and other societies catering to diverse migrant populations, or how best to account for clients’ religious beliefs and experiences in formal services provision. It is also unclear what models of collaboration between secular and faith-based providers in migrant and minority communities might be most effective currently. It should be noted that there is extensive evidence on programs engaging religious leaders in the context of GBV responses internationally (as summarized in Istratii and Ali, 2023), but these are usually funded or implemented by international development organizations with or without country partners in the context of international development aid or public health programming (e.g. Le Roux, 2017; Le Roux and Loots, 2017) and could not be defined as country-led organized DVA services.

With this gap as a starting point, we conducted a scoping literature review to explore and to identify current approaches used by DVA service providers, social workers, counsellors and other professionals working at the frontlines of responding to DVA or VAWG in the UK services sector and other societies where DVA services are organized through the state or charity sectors and engage extensively with migrant, ethnic minority or religious communities. A rapid scoping review was selected as an appropriate methodology given our aims to explore the scope and type of the available evidence and to inform further research. Our ultimate objective was to be guided by this evidence in order to contextualize and inform primary qualitative research with members of migrant communities and a sector-wide survey with DVA service providers in the UK. The review included all sources of evidence that referred to DVA support and services to victims, survivors and perpetrators from migrant and ethnic minority religious communities. In total, 30 publications were included and are discussed in this paper.

## Methods

### Definitions

In the context of this review, religious beliefs were understood as being rooted in the religious affiliation, socialization and worldview of a DVA victim, survivor or perpetrator, and faith-based resources were defined as resources available in religious communities and religious spaces, such as spiritual advice, prayer or mediation by religious teachers, clergy or other religious personnel. Recognizing that within health and psychological studies the term ‘spirituality’ has often been used interchangeably or in close association with ‘religion’ (Hodge, 2004; Marterella and Brock, 2008), we also included evidence that referred to integrating spirituality or spiritual beliefs in domestic violence services to the extent that this encompassed religious beliefs.

Moreover, since the concept of religion or faith has often been subsumed into the concept of culture, we also searched for studies that referred to ‘cultural sensitivity’, ‘cultural awareness’ or ‘cultural competence’ in domestic violence services that made direct reference to religious beliefs, faith or spirituality. We were particularly interested in understanding to what extent the literature on ‘culturally competent’ services, primarily emerging in North America and the UK where this concept and paradigm has been increasingly streamlined and debated, had accounted for religious beliefs and resources and had sought to conceptualize this relationship more systematically, but also how this might compare to approaches and understandings from across the world. See Tables 1 and 2 for full overview of included studies and materials.

**Table 1.** Included academic studies.

Author	Year	Country	Purpose statement
Kulwicki et al	2000	USA	Explores the perceptions, experiences, and patterns of health care behaviour among Arab Americans in an urban Midwestern area of the United States and the perceptions and experiences of health care providers related to culturally competent care
Burman et al	2004	England	Addresses how DVA services to women of African, African-Caribbean, South Asian, Jewish and Irish backgrounds are structured by assumptions about ‘culture’ which produce barriers to the delivery of domestic violence services
Hodge	2004	USA	Reviews Evangelical cultural narratives to develop cultural competency with evangelical Christians, the nation’s largest spiritual minority, providing context for a discussion of various practice interventions
Latta and Goodman	2005	USA	Explores how the cultural context of IPV affected accessibility to DVA services for Haitian women, as one immigrant group in the USA
Pan et al	2006	USA	Describes the Ahimsa for safe families project, an innovative collaborative project that addresses DVA in immigrant and refugee communities in San Diego and presents the design and culturally specific prevention strategies utilised by the project
Arnette et al	2007	USA	Investigates the ability of factors, such as level of hopelessness and the use of positive religious coping strategies, to predict spiritual well-being over time
Pyles	2007	USA	Draws on data from a study conducted in Wyoming about the community response to domestic violence to evidence how religion is paradoxically both a source of assistance and a barrier to women surviving DVA
Yick	2008	USA	Explores, extracts, and synthesizes themes from related qualitative studies on the role of spirituality and religiosity with culturally diverse DVA survivors
Gillum	2008	USA	Investigates how helpful a culturally specific IPV program, which targets the African American community, has been to African American female survivors
Gillum	2008	USA	Presents a study with African American survivors to explore (a) what their experience was with various community entities and (b) how they feel race may have affected these experiences
Gillum	2009	USA	Presents a study that was designed to ascertain, from African American survivors, what their experiences were with mainstream IPV interventions and how their experience with a culturally specific DVA agency was different from those experiences
Fowler et al	2011	USA	Presents a study that examined differences between shelter and faith-based service utilisation and satisfaction in a shelter sample
Bent-Goodley	2013	USA	Reviews current knowledge about cultural competence for Black women abused by men in the United States, with suggested implications for domestic violence fatality review teams (DVFRTs)
Stennis et al	2017	USA	Describes the development of the S.T.A.R.T.© education and intervention model, a religiously-sensitive and spiritually-based, multi-dimensional IPV education and intervention model
Jayasundara et al	2017	USA	Presents the main premises of Buddhism, Christianity, Hinduism, Islam and Judaism and discusses how each is separately misused to justify abusive behaviour, and common social work practice strategies that can be used in DVA situations with all religions
Ghafournia	2017	Australia	Explores the role of religious values in Muslim migrant women’s experience of IPV and implications for faith-based prevention and intervention strategies in the Muslim community
Crisp et al	2018	Australia	Explores what religious literacy means in regard to protecting children from sexual assault in Australia’s Jewish community and Muslim women who experience DVA
Milani et al	2018	Canada	Examines frontline service providers’ perspectives on Muslim women’s experiences of IPV
Mulvihill et al	2023	UK	Explores the lived experiences of coercive control drawing on victim and witness accounts of abusive intimate partner relationships across Christian, Muslim, Jewish, Sikh and Buddhist faith contexts in the UK

**Table 2.** Included grey literature and organisational publications.

Title	Author	Year	Country	Purpose
Faith, immigration and domestic violence: Concepts for working with faith communities	National council of juvenile and family court Judges (Author: Kelly Ranasinghe, Esq)	2020	USA	Provides introductory guidance on the intersection between faith, immigration, and DVA intended for both advocates and court professionals
Domestic violence and faith communities: Guidelines for leaders	Office for the prevention of domestic violence with help for New York Theological Seminary	no date specified	USA	This document is designed to assist faith leaders in responding to DVA within their communities by providing guidelines and information about working with individuals who are abused, and by encouraging them to raise awareness of the issue within their communities
Chapter 12: Engaging religious, spiritual and faith-based groups and organizations	National Advisory council on violence against women	2004	USA	Outlines specific actions religious, spiritual, and faith-based organisations, community-based sexual assault and DVA programs, secular victim services, advocacy programs, and public and private funders can take to end violence against women
Faith and domestic abuse: Recommendations for faith leaders	Faith action	2015	UK	A resource that provides recommendations to faith leaders on how to respond to DVA in their communities
Creating a safe place: Encourage to change family peace-making Materials for clergy, lay leaders, staff & Laity	Anoka County faith community	2000	USA	Course content and tools that teach DVA awareness and the role of clergy and lay leaders intended to be co-taught by a representative of the faith community and a local advocacy services community educator
Faith and intimate partner violence handbook for advocates	Florida Coalition against domestic violence and faith trust institute	2017	USA	A handbook intended for advocates to engage with religious beliefs/faith in their work as part of building cultural competency
Keeping the faith what survivors from faith communities want us to know	Faith & VAWG Coalition (authors: Huda Jawad and Zainab Moallin)	2020	UK	A report that seeks to uncover the unique challenges and experiences related to COVID-19 for women from faith communities and to inform policy directions
Religion and domestic violence: Information and resources	National resource center on domestic violence (NRCDV)	2007	USA	A collection of information packets to serve as an introduction to the complex and varied issues that religion and faith can present for DVA victims and survivors and their advocates in both the faith and secular communities
Domestic violence Factsheet for Service providers	Peaceful families project (author: Hasnaa Mokhtar)	n.d	USA	A leaflet/one-page guidance that instructs service providers on how to respond to DVA in Muslim communities
Responding well to domestic abuse: Policy and practice guidance second edition	The church of England	2017	UK	The church of England policy on DVA training and responses in parishes
A commentary on religion and domestic violence	Faith trust institute (authors: Rev. Marie M. Fortune, Salma Abugideiri and Rabbi Mark Dratch)	2010	USA	Provides a discussion of the basic understanding of the place of religion in addressing DVA, illustrated through three western religious traditions: Christianity, Judaism, and Islam
Executive summary: Evaluation of CRDVN (culturally responsive domestic violence network) final report	Social policy research (SPR)	2018	USA	Provides a summary of the evaluation findings of CRDVN, a community-driven approach to building the overall capacity of the DVA field to prevent and address DVA among all Californians
Culturally responsive intervention: Summary of evidence	DRIVE partnership (author: Elaha Walizadeh)	2022	UK	A review on culturally responsive interventions for perpetrators of GBV. Outlines the findings and recommendations of the review

### Search protocol

The protocol of the review was developed collaboratively among the authors of this paper, who combined different backgrounds and specializations, including health/public health (Gene Feder; Parveen Ali), religious studies, anthropology and international development (Romina Istratii), and experience working in DVA services in the UK (Linda Mshweshwe). The searches, data analysis and synthesis were completed by RI, with LM mapping organizational literature and supporting data extraction, and GF and PA advising throughout the process and contributing to the final report.

A scoping review methodology was selected as appropriate to determine the state of the evidence before undertaking more rigorous empirical research (Constand et al., 2014), which was our ultimate aim. The review broadly followed the principles outlined in Arksey and O’Malley’s (2005). This approach comprises of five stages involving defining the research question, identifying relevant studies, selection, charting the data and collating, summarizing, and reporting the results (Arksey & O’Malley, 2005). The rapid scoping review involved: (a) qualitative or quantitative studies, (b) literature reviews, including systematic reviews, (c) relevant studies or grey literature published by DVA or VAWG organizations, other relevant charities, government agencies working on DVA, or professionals who are offering services through organized DVA services led by the state or charity sectors. We also reviewed the bibliography lists of papers that we identified. Relevance was decided based on title; where this was ambiguous, we also read the abstract of the papers considered relevant. If the abstract addressed questions overlapping the aims of this review they were read in full.

As we were reviewing evidence, we excluded conceptual and planning papers, but included grey literature to capture the non-academic evidence produced and published by DVA service providers. All the resources reviewed had to be in English to be accessible to all the members of the team and to have been published in the 21^st^ century, although grey literature that was found to be relevant was included even when no date of publication was reported.

The review included all sources of evidence that referred to DVA support and services to victims, survivors and perpetrators from migrant and ethnic minority religious communities. We did not employ a rigid definition for the category ‘migrant’ or ‘ethnic minority’ and sought to include communities for whom religious beliefs were evidenced to be important in domestic violence experiences and help-seeking. Since we were interested in migrant communities and the cross-cultural context, we did not search for evidence on DVA organized responses or services in majority religious communities based in their home countries.

Study settings could be statutory domestic violence services, women’s or VAWG organizations or charities supporting survivors, including psychological support or social care integrated in frontline DVA services, as well as ‘by and for’ initiatives (led by or focused on specific cultural or religious communities) that were integrated into the organized sector. The review also captured service providers’ engagements with perpetrators, such as through perpetrator treatment programs. Conversely, the review excluded studies of programs that did not directly address their links to organized DVA services.

The review also excluded: (a) studies that referred to the effect of faith and religious beliefs on the help-seeking of DVA survivors and perpetrators, which had already been covered in a previous literature review (Istratii and Ali, 2023), unless they made direct recommendations and had implications for faith-sensitive DVA services; (b) studies that discussed faith-sensitive interventions in community settings without discussing the perspectives of DVA service providers, as this review was specifically interested in the approaches and experiences of service providers and their types of engagement with religious beliefs and faith-based resources, and (c) studies focussing on culturally competent services unless these made explicit reference to religious beliefs and faith or faith actors.

Searches were run by RI using Google, Google Scholar and EBSCO search engine for social sciences and humanities. Titles and abstracts were searched using keyword combinations as listed below: Religion/Faith AND Domestic Violence Services/Provision/Support; Religious sensitivity AND Domestic Violence Services/Provision/Support; Cultural sensitivity AND Domestic Violence Services/Provision/Support; Religious competence AND Domestic Violence Services/Provision/Support; Cultural competence AND Domestic Violence Services/Provision/Support; Religious Literacy AND Domestic Violence Services/Provision/Support; Cultural Literacy AND Domestic Violence Services/Provision/Support; Service Provider Perspectives AND Religion/Culture; Service Provider Perspectives AND Spirituality; Domestic Violence Providers AND Spirituality; Domestic Violence AND Cultural Competence; Domestic Violence Services AND Religious Competence; Domestic Violence Services AND Cultural Competence; Domestic Violence Services AND Religious Literacy; Domestic Violence Services AND Cultural Literacy; Domestic Violence Providers and Religion; and Domestic Violence Services AND Cultural Competence. The term ‘domestic violence’ was also replaced with the more specific term ‘Intimate Partner Violence’ as the most frequent form of domestic violence that has attracted extensive scholarly attention.

In our early stages of building the review protocol, we observed that the term ‘religious literacy’ was at times used by organizations promoting faith sensitivity in the sector, but the term was rarely clearly defined. In the current literature review, we were therefore interested to identify existing definitions and understandings of this term. We chose to use it as a search word precisely because it has been increasingly used in the DVA sector, albeit without rigorous theoretical context.

**Figure 1.**
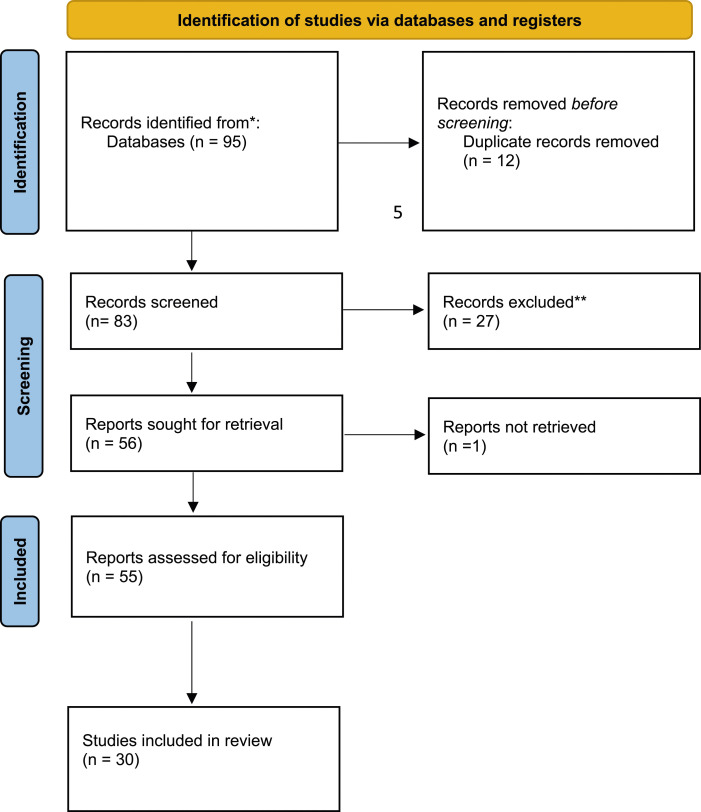
PRISMA flow diagram for systematic review. From: Page et al. (2021).

Additional searches were run that explored issues of racism, colonialism, decolonization, diversity and social justice in VAWG and DVA services provision as it was anticipated that these could overlap with questions of cultural sensitivity and religious diversity. Moreover, the review team conducted a systematic search of grey literature from DVA service providers and VAWG organizations in the UK. To capture as much possible of this grey literature, in addition to RI running searches on Google, LM mapped major relevant stakeholders and service providers in the UK and reviewed their websites for related content. The stakeholder list included 90 organizations with available website and online presence. The results of the review are presented in a PRISMA graph below Figure 1.

## Findings

The papers and materials that met the inclusion criteria listed earlier were organized in six evidence categories:

(a) Category 1: Papers engaging religious communities and religious survivors of DVA that presented a rationale or recommendations for integrating religious beliefs and faith-based resources in services supporting migrant or ethnic minority communities.
(b) Category 2: Papers that presented a rationale or an approach for culturally competent or culturally appropriate services that also mentioned religious beliefs, faith or religious stakeholders in the recommendations of their papers and in suggesting needs for future research.
(c) Category 3: Papers that sought to apply a faith-sensitive lens to DVA services working with religious communities or that evaluated programs that had followed such an approach.
(d) Category 4: Materials that focused on improving the preparedness of religious leaders to support survivors and perpetrators with safety concerns in mind and with awareness of available services, developing religious literacy among DVA providers, or promoting cross-sectoral collaborations between religious stakeholders and domestic violence providers.
(e) Category 5: Studies that referred to faith-sensitive approaches in psychotherapy and counselling to DVA survivors and perpetrators.
(f) Category 6: Papers that intersected the study of domestic violence services with issues around the concepts of culture, racism and religion.

### Papers that recommended inclusion of religious beliefs in DVA services

Many of the reviewed studies examined the influence of religious beliefs and mediators in the DVA experience of victims or survivors and their help-seeking, making direct recommendations to inform domestic violence services (Fowler et al., 2011; Ghafournia, 2017; Mulvihill et al., 2023; Pyles, 2007; Yick, 2008). Most of these studies were from the USA, one was from Australia and one from the UK.

A study from the USA that drew on data collected in Wyoming to assess community responses to DVA (Pyles, 2007), scrutinized the role of religion and found this to have served both as a resource and a barrier. While religious institutions could provide emotional comfort and practical assistance, the response of religious personnel could also perpetuate silence. The study found limited collaboration between religious institutions and social service providers and recommended that better links be developed between religious spaces and secular services.

Yick (2008) explored the role of spirituality and religiosity in domestic violence experiences of diverse women, placing central attention to the influence of their culture-specific worldviews in the intersection of spirituality and DVA. The study included survivors from primarily the African American community, with a few being Asian women, White women and other ethnic groups. In general, faith and invoking a higher power helped the women to cope, while the existence of religious institutions and resources was identified as a source of strength. At the same time, tensions existed between the women’s own abusive relationships and how their religious traditions perceived marriage and gender roles. Based on the findings, the author proposed that practitioners should explore how their clients’ religious beliefs and spiritual experiences might influence their ways of coping with the understanding that a woman’s religiosity or spirituality is influenced by the environment around her, her community and her family. Therefore, practitioners should collaborate with faith communities and religious institutions to provide culturally competent services, suggesting thus a conceptualization of cultural competence that integrated faith sensitivity

A study engaging the Muslim community in Australia reinforced these findings (Ghafournia, 2017). The study engaged 14 abused women to explore the role of religious values in Muslim migrant women’s experience of IPV and implications for faith-based prevention and intervention strategies. In contrast to stereotypes about Muslims, the study found that women in the study turned to their religious beliefs as one of their main strategies in dealing with the abuse, although the response of religious leaders was described mostly negatively. Important insights emerged also in relation to how women viewed the role of Islam and religious beliefs in their abuse, and how they interpreted negative attitudes in relation to cultural systems and socialization. Perpetrators used religious language to justify their abusive behaviour, suggesting that a patriarchal interpretation of Islam and certain cultural elements contributed to women’s oppression (Ghafournia, 2017: 159). The paper concluded that a faith-informed framework could be used to identify appropriate intervention strategies. To be able to do this, however, social workers should develop a basic understanding of Islamic tenets to navigate abused women’s invocations of Islam and to support them without bias.

In one of the few studies reviewed based in the UK, Mulvihill et al. (2023) explored the lived experiences of coercive control drawing on victim IPV accounts across Christian, Muslim, Jewish, Sikh and Buddhist faith contexts in the UK. The analysis was based on two multi-faith datasets: secondary data analysis of 27 semi-structured interviews conducted over 2016–2017 and primary data collected through an online anonymous survey in 2021 eliciting 24 qualitative responses, supplemented by limited interviews with victim-survivors. The authors discussed the concept of spiritual abuse as developed over two decades in the UK and drew attention to the use of distorted religious language by perpetrators to control their partners, which the authors called ‘religious coercive control’. The study proposed that survivors of religious coercive control may need specialist support to address and overcome spiritual trauma. One implication of the study was that religious spaces must ensure that training is provided to support and safeguard survivors and that they serve as a key component in the ‘social web of accountability’ to respond to IPV.

Relevant to this discussion is a study that investigated the effect of spirituality in services utilization for women residing in a domestic violence shelter in the USA (Fowler et al., 2011). The study defined spirituality as a way of being, meaning-seeking around the purpose of life and interaction with a higher power. The study found that women with higher levels of spirituality were less likely to have utilized shelters and more likely to have utilized faith-based resources. According to the findings, survivors with higher spirituality were more likely to utilize faith-based resources than shelters, but those survivors who experienced greater IPV reported dissatisfaction with faith-based resources. The results suggest that spirituality should be incorporated into shelter services to meet survivors’ spiritual needs, and that faith-based services should adequately address IPV by collaborating more effectively with DVA services.

### Papers that presented a rationale or an approach for culturally competent or culturally appropriate services that integrated religious beliefs or religious resources

The literature on culturally competent or responsive approaches is extensive, with the paradigm of ‘cultural competence’ appearing to be more established in North America and Canada (Congress, 2005; Danso, 2018; Hernandez et al., 2009; Jackson et al., 2015; Klingspohn, 2018; Messing et al., 2013; Pokharel et al., 2021; Powell Sears, 2012; Rana, 2012; Sumter, 2006; Whitaker et al., 2007; Wretman et al., 2022), but gaining critical attention in the UK too. The studies in this category included religious beliefs, faith or religious mediators in their definitions of culturally appropriate services, or in the implications of their findings and future directions (Bent-Goodley, 2013; Gillum, 2008a; Kulwicki et al., 2000; Latta and Goodman, 2005; Walizadeh, 2022).

Many papers highlighted the need to develop a proper understanding of community context, the role of family, religious leaders or other influential figures in shaping victims’ help-seeking attitudes, and standards, norms and expectations about family life, marriage and domestic violence. In a paper that reviewed knowledge about cultural competence for Black women abused by men in the USA, Tricia Bent-Goodley (2013) reported that the religious community was generally considered a resource for African American domestic violence victims, however, faith-based providers often gave unhelpful advice or inadequate help. Based on a story of Yolanda, a woman killed by her husband who had sought refuge in her faith, the author stressed the need for contextual analysis to understand views about domestic violence upheld in the community and structural barriers experienced by victims. The author also noted the potential role of churches, civil organizations, women’s ministries and other community-based organizations in support and prevention efforts.

Another study investigated how the cultural context of IPV affected accessibility to mainstream DVA services for Haitian women in the USA (Latta and Goodman, 2005). The authors found that immigrant women hesitated to seek support from mainstream services because they failed to understand their context-specific IPV experiences. The paper also found that Haitian women often resorted to pastors and faith leaders for advice, who generally were unsupportive. Based on these findings, they proposed that faith leaders should be trained to be able to direct victims and survivors to relevant services and to help raise awareness and change community norms in culturally appropriate ways.

A study by Kulwicki et al. (2000) that took place in an urban Midwestern area of the USA investigated the patterns of health care behaviour among Arab Americans and attitudes among service providers, stressing the need for cultural sensitivity. For example, one Arab service provider complained about stereotyping all Arabs as Muslim, speaking about the need to enhance religious literacy to avoid generalizations. The authors found that while providers placed emphasis on ‘same treatment for all’ in response to concerns of racism and discrimination, members of the community favoured the need for cultural sensitivity, raising the need for a closer engagement with client’s understandings.

Gillum (2008a) in turn, explored the experiences of African American survivors with various community entities. The study entailed focus groups and individual interviews with 13 African American female survivors of DVA and two African American female service providers. It reported that women were generally dissatisfied with services, especially when trying to leave or stay away from an abusive partner. The reasons were related to cultural incompetence and racism, such as services lacking African American staff and not providing a culturally safe environment. The study participants also believed that churches could play an important role in battling IPV in their congregations by condemning it more openly and offering support groups to assist survivors, in lieu of couple counselling that was believed to rather couch the issue of IPV in the community.

In the UK context, Walizadeh’s (2022) review of culturally responsive interventions for perpetrators of GBV directly addressed the integration of religious parameters in culturally competent responses, proposing that there is a need to overcome Eurocentric ideas about ‘religion’. While the review did not list specific studies on the integration of religious beliefs in cultural competency programs, it found that the influence of ‘culture’ or ‘religion’ was often ignored in DVA work even though ethnic minorities and migrants were disproportionately represented in refuges. The review also proposed the necessity to integrate a more intersectional lens on DVA to account for diverse needs, colonialism and intergenerational factors. Lastly, the review, recognizing that cultural competency could be appropriated by mainstream DVA service providers, stressed that it should not be used as excuse to minimize the work of services ‘by and for’ members of specific cultural communities.

### Papers that sought to apply a faith-sensitive lens to DVA services working with religious communities or that evaluated such programs

Studies and resources under this theme could be divided into two types: studies that presented the particular characteristics of communities of faith with the aim of improving religious literacy among social workers and DVA providers (Hodge, 2004; Jayasundara et al., 2017), and studies that presented approaches integrating a spirituality component in culturally competent services catering to migrant, ethnic or religious minorities, or that compared the benefits of culturally specific versus more generalist services (Gillum, 2008b, 2009; Pan et al., 2006; SPR, 2018; Stennis et al., 2015). Overwhelmingly, this evidence comes from the USA, although it also included a study from Australia that explored religious literacy in protecting children from sexual assault in Australia’s Jewish community and in responding to Muslim women who experience DVA (Crisp et al., 2018).

In one of the earliest papers reviewed, Hodge (2004) presented central aspects of evangelical Christians’ cultural narratives around gender relations, marriage and DVA addressing service providers. Hodge proposed that service providers needed to build a better understanding of the religious worldview of this community, such as by reading family resources written by authoritative Evangelical Christian authors. Hodge recognized that believers could manifest their spirituality in harmful ways and advised that social workers should not attempt to problematize the veracity of their clients’ theological beliefs but could put them in touch with a pastor. Concerned about misrepresentations of evangelical Christians in the mainstream, Hodge stressed the importance for service providers to reflect on any personal biases or stereotypes and even argued that where the values of the social workers and the clients fundamentally differed, it would be ethically compelling to redirect them to providers who may be better placed to cater to their needs.

Another paper published in the USA (Jayasundara et al., 2017) discussed five major religions ― Buddhism, Christianity, Hinduism, Islam and Judaism ― and analysed how religious teachings could be misused to justify abusive behaviour. The authors explained their rationale for placing emphasis on religious tenets referring to how they shape gender roles, sometimes reinforcing gender inequality in the context of literal interpretations of sacred texts (Jayasundara et al., 2017: 41). *Inter alia*, the authors called for social workers to have basic knowledge of their clients’ religious traditions to be able to help them ‘explore alternatives that counter these misinterpretations’ (58). Crisp et al. (2018), in turn, explored what religious literacy might mean in the context of protecting children from sexual assault in Australia’s Jewish community and in supporting Muslim women who experience DVA. The aim of the paper was to show students and practitioners in social work and healthcare the importance of engaging carefully with their clients’ religious beliefs. While the authors acknowledged that in Muslim-majority countries there is often the perception that DVA is condoned within Islam, they stressed that the problem reflected the underlying issue of women’s unequal status to men. Moreover, opinions about DVA and Islam can be more diverse than it is often recognized and include Islamic feminist scholars’ activism and abused women’s own conditions and understandings. This stressed the importance of understanding the religio-cultural contexts and standards upheld in the communities of female survivors of DVA, avoiding generalizations.

The review included also a limited number of studies that described approaches that sought to be appropriate to religious and cultural context, such a paper presenting the Ahimsa for Safe Families Project set up to address DVA in immigrant and refugee communities in San Diego, USA (Pan et al., 2006). Ahimsa, which means nonviolence or non-harm in Sanskrit, was designed to increase awareness of DVA among Latino, Somali and Vietnamese communities relying first on a needs assessment of their respective attitudes and beliefs about DVA and then holding community dialogues to discuss the findings. The project developed and implemented culturally specific programs targeted at each of the three communities, which evidenced the importance of contextualizing DVA within the cultural socialization of the community, pointing also to important overlaps between religious tradition and identity.

Community-based needs assessment identified six core issues underlying DVA, which were: varying definitions of violence, family harmony, strict gender roles, conflict resolution strategies, cultural identity and spirituality (42) and numerous barriers that could hinder women from accessing services, such as a lack of trust, language barriers, transportation, beliefs about family/culture, and a lack of bilingual/bicultural staff. The authors concluded that implementing a culturally competent response would necessitate understanding the cultural values of the community and how these can influence the behaviour of DVA victims, survivors, perpetrators, their families and the community. They also stressed the need for service providers to approach diverse communities with respect and sensitivity.

Another spiritually informed, culturally competent program that was identified in the reviewed literature was the S.T.A.R.T.^©^ Education and Intervention Model in the USA (Stennis et al., 2015). The model was developed as a religiously sensitive and multidimensional IPV education and intervention model for the African American faith community in the USA. START, which stands for Shatter the Silence, Talk About It, Alert the Public, Refer, and Train self and others, has worked to empower individuals to assess beliefs around power, gender, types of abuse, individual and community responsibility and refer those affected to available resources. Since its inception, the model has been implemented in numerous communities, including African Americans, Hispanic religious leaders and Christian and Muslim Ethiopian women’s advocates, and has been assessed using a post-training focus group format. The overall feedback and evaluations received have been positive, with participants appreciating the religious diversity addressed in the model, the culturally sensitive content, and the ease of using it for discussing sensitive topics, including sexual exploitation. The authors also proposed some implications for Christians in competency-based social work practice, observing the need for practitioners to consider how their own values relate to the communities they cater to, and actively building on the religious, cultural and spiritual values of their clients without losing sight of the diversity that exists within faith communities of colour.

A third example is the Culturally Responsive Domestic Violence Network (CRDVN) set up in California, USA (Social Policy Research SPR, 2018) to better respond to DVA among immigrants and communities of colour. The evaluation of the network identified that three CRDVN partners had engaged faith-based leaders, through different strategies, which included: adopting a humble approach to partnership, training faith leaders as first responders to DVA, and developing alliances across different faith communities. The authors concluded that engaging clergy and churches was effectively used to promote culturally responsive approaches and to shift community norms around DVA.

In another study published in the USA, Tameka Gillum (2008b) investigated how helpful a culturally specific IPV program targeting the African American community had been to female survivors. The research site was a culturally specific DVA agency, located in a mid-sized Midwestern city, catering to African American community. The study involved interviews with the coordinator of Victim’s Services, two of the agency’s women’s advocates, and 14 service users. A spirituality-based approach was identified as a culture-specific element in the agency’s work, as well as an Afrocentric curriculum, an Afrocentric environment, a holistic and family-centred approach, and staff representation. The results indicated the success of culturally specific interventions with African American survivors and highlighted the need for more programs of this nature. Additionally, the survivors found the agency’s approach helpful because it did not force spirituality on those who were not interested and embraced spirituality in a non-denominational way.

In a follow-up paper, Gillum (2009) investigated African American survivors’ experiences with mainstream IPV services. While these were reported to have somehow helped, such as providing a safe environment and basic resources, the women described mostly problematic experiences with those providers, which contrasted with the more positive feedback on the culturally specific agency. Mainstream services were described as inadequate under the following themes: (a) the culture of the organization was not welcoming to African Americans, (b) insensitivity to the process of leaving an abusive relationship, (c) barriers to adequate assistance, and (d) a non-supportive environment. The culturally specific agency was found helpful especially because it understood the women’s complex and multiple needs, particularly when leaving an abusive relationship. A survivor also mentioned that they did not like that one mainstream service provider promoted Catholicism, a faith that they did not share. In recognition of the fact that not all services can become culturally specific, the authors suggested that mainstream services should aim to become more culturally specific in their recruitment strategies and intervention approaches.

### Resources that focused on improving the preparedness of service providers and religious leaders to support religious survivors and perpetrators, or promoted cross-sectoral collaborations between religious stakeholders and service providers

A large evidence category comprised of organizational publications in the form of guidance sheets, toolkits and manuals targeted at religious stakeholders and DVA service providers. Some aimed to improve religious leaders’ responsiveness to victims, survivors and perpetrators in line with safeguarding standards and in collaboration with generalist and secular DVA services. Others focused on developing the ability of secular service providers to engage with the religious beliefs of their clients and collaboration with religious stakeholders. A third group of resources addressed both faith leaders or faith-based organizations and DVA service providers, with the aim of promoting mutual understanding and collaboration. Eleven such resources were retrieved in the search, but these are certainly not exhaustive.

Echoing the academic literature, the grey literature reviewed seemed to uphold that faith could become a resource in responding to DVA. Additionally, reiterating more academic propositions that religious beliefs should be integrated in culturally competent services, some resources employed the concept of cultural competence in tandem with spirituality as practice of faith, seeing it is an important aspect of ‘culture’.

### Resources for religious leaders to improve their awareness and responsiveness to domestic violence

One such example is a guidance sheet developed by the Office for the Prevention of Domestic Violence in association with the New York Theological Seminary (n.d.) in the USA titled *Domestic Violence and Faith Communities: Guidelines for Leaders*. This aims to assist faith leaders in responding to DVA within their communities. The relationship between the concepts of culture and religion is also discussed, suggesting that the problem often lies in ‘religion’ being used by ‘culture’ in negative ways. Another inter-faith manual with similar objectives was published by Faith Action (2015) in the UK titled *Faith and Domestic Abuse: Recommendations for Faith Leaders*. The guidance takes an inter-faith approach, listing specialized materials for Christian, Muslim, Jewish, Hindu and Sikh faiths, but does not consider in more depth theological differences within these communities.

Other organizations and initiatives have developed or offered course materials and toolkits to build clergy responsiveness, such as the training guidance published Anoka County Faith Community (2000) titled *Creating a Safe Place: Encourage to Change Family Peacemaking Materials for Clergy, Lay Leaders, Staff & Laity*. The course content and tools provided seek to raise awareness of DVA among faith leaders in Christian congregations and among lay community members. The course was designed to be co-taught by a representative of the faith community and a local advocacy services community educator.

The reviewed grey literature included also policy documents developed by religious institutions that identified how their personnel should respond to DVA within their congregations. An example is the Church of England’s policy on domestic abuse training and responses in parishes developed by the House of Bishops (The Church of England, 2017). *Inter alia*, the policy sees the clergy as responsible for facilitating a multiagency response to DVA and identifies measures that should be taken at diocese and parish-level. The policy includes instructions also for licensed lay ministers.

### Resources for DVA providers and advocates to engage with religious leaders and beliefs

A characteristic example of such a resource is the manual *Faith and Intimate Partner Violence Handbook for Advocates* developed by the Florida Coalition Against Domestic Violence and FaithTrust Institute (2017) in the USA for advocates to engage with faith in their work. This is one of the few manuals that employ the concept of cultural competence in tandem with spirituality, which it defines as the practice of faith. This guidance encourages advocates to take an exploratory approach and discuss with the client the importance of faith and religious resources in their life. The manual centres on self-examination of service providers’ positionality and the possible biases this may engender. Moreover, the manual refers to spiritual abuse as a form of abuse that advocates should familiarize themselves with.

Along the same lines, the guidance *Religion and Domestic Violence: Information and Resources* produced by the National Resource Center on Domestic Violence (NRCDV) (2007) in the USA proposes that faith can become a resource in responding to DVA. By being sensitive to their clients’ religious beliefs, service providers can help them to identify relevant options and resources. The manual also discusses the limitations and biases that a secular provider might have about faith communities, encouraging more genuine collaborations with faith leaders.

Lastly, the guidance sheet titled *Faith, Immigration and Domestic Violence: Concepts for Working with Faith Communities* authored by Ranasinghe (2020) on behalf of the National Council of Juvenile and Family Court Judges in the USA provides an introduction on the intersection between faith, immigration and DVA for advocates and court professionals. This guidance explains and stresses the importance of ‘religion’, which it places within a community’s larger cultural identity, in DVA experiences. It recommends working with faith leaders as an important way of integrating a faith-sensitive perspective.

### Resources that address both faith leaders and DVA service providers

One example of such a resource is chapter 12 of the Toolkit to End Violence Against Women developed by the National Advisory Council on Violence Against Women (2004) in the USA titled *Engaging Religious, Spiritual, and Faith-Based Groups and Organizations.* The 5-page guidance emphasizes collaboration with religious leaders in DVA responses and outlines specific actions for religious organizations, community-based and secular victim services, advocacy programs and public and private funders.

Another example is the brief *Keeping the Faith: What Survivors from Faith Communities Want Us to Know* produced by Jawad and Moallin (2020) on behalf of the Faith and VAWG Coalition in the UK, which is focused on COVID-19 experiences among women from faith communities and advocates for a better integration of religious resources. This report addresses both faith leaders and state DVA services to help them understand the importance of engaging with faith reflectively. *A Commentary on Religion and Domestic Violence *authored by Fortune et al. (2010) on behalf of the Faith Trust Institute in the USA similarly tries to improve religious literacy in the DVA sector, addressing both religious leaders and DVA service providers. The resource illustrates the importance and role of religious beliefs and faith-based resources in addressing DVA within Christianity, Judaism and Islam, with some components being faith-specific and others having cross-religious relevance.

The materials that were viewed included also a *Domestic Violence Factsheet for Service Providers* authored by Mokhtar (n.d.) on behalf of the Peaceful Families Project, in the form of a one-page guidance that instructs service providers how to respond to DVA. The guidance addresses Islamic faith leaders (imams, kahteebs, others working in mosques) and shares instructions on what they should do when they reference the Qur’an/hadith in their teachings.

### Papers that referred to faith-sensitive approaches in psychotherapy and counselling to DVA survivors and perpetrators

The current review identified only one study that related spiritual factors to coping and healing for DVA survivors from ethnic minority communities in the context of DVA services specifically. The one study included in the current review (Arnette et al., 2007) seemed to reinforce evidence pointing to the importance of integrating religious coping strategies in culturally appropriate services to religious minorities (Istratii and Ali, 2023).

Arnette et al. (2007) investigated the ability of factors, such as level of hopelessness and the use of positive religious coping strategies, to predict spiritual well-being over time. The study took place at a trauma hospital in the USA and collected self-report questionnaires measuring hopelessness, use of religious coping strategies, and two domains of spiritual well-being from 74 survivors of IPV who had attempted suicide within the prior year. Spiritual well-being was defined to incorporate both existential meaning and relationship to God and was measured by the Spiritual Wellbeing Scale. The study found that lower levels of hopelessness predicted increases in existential well-being over time, while higher levels of positive religious coping predict increases in religious well-being over time. Since spiritual well-being is associated with better psychotherapy outcomes for African American women, and since hopelessness and religious coping predict spiritual well-being, an argument could be made for attending to these predictors when implementing culturally relevant interventions with abused, suicidal African American women.

### Papers that intersected the study of domestic violence services with issues around the concepts of culture, racism and religion

One of the fewer papers published on the topic in the UK (Burman et al., 2004) drew from a previous study to explore how DVA services to women of African, African-Caribbean, South Asian, Jewish and Irish backgrounds were structured by assumptions about ‘culture’, which they argued could produce barriers for the delivery of DVA services. The unique value of this paper is that the authors focused on the intersections of mainstream (dominant culture) services and minoritized community responses to explore the cumulative effects on women from minority communities. They identified two mechanisms that led to the marginalization of minority women’s abuse, tendencies that resembled cultural relativism and the pathologizing of minority communities.

The paper also discussed how different providers, mainstream and culturally specific, engaged with cultural differences. These organizational accounts of support for minoritized women were classified according to four discursive strategies: ‘it’s all the same’; ‘softly, softly’; as mediated by ‘cultural privacy’ via a discourse of ‘cultural respect’; and via discourses of professional specialization. Within dominant culture services, emphasis was placed on gender roles and inequalities between men and women, and the nuclear heterosexual relationship, which did not always match the experiences of minoritized women and the structural, racial and community factors defining the experience of DVA. Other organizations, including the police, did not interfere out of ‘cultural respect’ or out of a fear or being perceived as racist, which could contribute to women’s abuse not being recognized. On the other hand, culturally specific organizations often worked ‘softly softly’ with the support and through community leaders. While this strategy was found less stigmatizing by women, survivors’ accounts suggested that such organizations did not offer an arena to speak openly about DVA.

The authors, ultimately, identified as problematic a segregation and compartmentalization of ‘specialisms’ within DVA statutory and charity services around the concept of ‘culture’, resulting in limited interventions due to providers lacking sufficient expertise. On the other hand, culturally specific organizations were reported to lack expertise in DVA interventions. Based on these findings, the authors called for a ‘both and’ approach to the delivery of DVA services that would involve both culturally specific services and mainstream services and suggested responding to minoritized women’s DVA needs with an understanding of how cultural, gender, racial and systemic factors combine to influence service providers’ reach and partnerships with others in supporting survivors.

Generalizations and stereotypes about certain communities, or tendencies to pathologize them as inherently violent were also consistently raised in the literature, as suggested in a study from Canada that examined frontline service providers’ perspectives on Muslim women’s experiences of IPV and women’s utilization of services (Milani et al., 2018). One implication was that service providers needed to be aware of the diverse nature of IPV in different societies to respond in culturally congruent ways without essentializing certain cultures and isolating cultural understandings of religious standards from wider sociological systems.

## Summary and discussion of the results

The majority of studies reviewed were based in the USA, where religious beliefs and spirituality have been more extensively recognized as an important parameter of cultural diversity for at least the past two decades (Bent-Goodley, 2013; Hernandez et al., 2009; Jayasundara et al., 2017; Kulwicki et al., 2000; Messing et al., 2013; Rana, 2012; Stennis et al., 2015; Sumter, 2006; Whitaker et al., 2007; Wretman et al., 2022). A smaller number of studies based in Australia engaged with Muslim and Jewish communities (Crisp et al., 2018; Ghafournia, 2017), while Canadian studies focused on Muslim and indigenous populations (Jackson et al., 2015; Milani et al., 2018). Two recent studies that explored the integration of religious beliefs and spiritual factors in DVA responses were based in the UK (Mulvihill et al., 2023; Walizadeh, 2022), with one older study addressing the intersection of culture, DVA services and racialization in minoritized communities (Burman et al., 2004). The review by Walizadeh (2022) was the only study we identified that attempted to systematically review culturally competent programs working with DVA perpetrators.

Many authors made an argument for a better integration of clients’ religious beliefs in DVA services and sought to improve service providers’ religious literacy (Crisp et al., 2018; Hodge, 2004; Jayasundara et al., 2017), or presented and assessed faith-informed or spirituality-centred culturally competent programs (Pan et al., 2006; SPR, 2018; Stennis et al., 2015). This evidence suggests that practitioners may need to educate themselves on different religious traditions to develop a rudimentary understanding of their tenets, but they should also recognize the variable ways in which religious traditions may have been expressed and embodied in different geographical and cultural contexts, informing DVA experiences and help-seeking attitudes in distinct ways. Providers should not lose sight of the diversity of experience that often exists within the same religio-cultural community and should try not to isolate cultural understandings of religious standards from wider sociological systems and gender structures and hierarchies (Milani et al., 2018). In view of the challenge to address these needs effectively, DVA service providers are advised to foster closer links with religious mediators and clergy who can offer authoritative and context-informed support.

The grey literature that was reviewed either placed emphasis on training clergy to respond better to DVA survivors and signpost them to available services or focused on building religious literacy among service providers themselves. Despite the nuanced understanding that underpinned many of the manuals and toolkits reviewed, the majority tended to fall into generalizing patterns that the academic literature cautioned against. This became evident in many resources being organized under the broad categories of Christianity, Islam, Judaism or other religious traditions, paying less attention to cultural context and the diversity of religious experience cross-culturally, and even within the same faith community.

Studies that specifically examined what made culturally competent services effective to minority communities (Gillum, 2008a; 2008b, 2009) suggested that DVA service providers needed to consider the needs and conditions of religious clients and proposed that religious spaces could offer targeted support to victims and survivors. Other studies pointed to the necessity for specialist services to respond to victims and survivors of spiritual abuse and trauma (Mulvihill et al., 2023). In responding to DVA to religious minority communities, both general and culturally specific services may be needed, but more consideration should be given to their interaction. As suggested above, generalist DVA services may need to become more aware about and responsive to the specific needs, conditions and socialization backgrounds of their diverse clients. Additionally, more culturally specific and faith-informed support initiatives may have to be integrated within the DVA generalist sector so that together they can reach a larger number of ethnic and religious minority communities living in western industrialized societies such as the UK.

In appraising these results, it is important to consider two caveats. While the evidence from Euro-America suggests a substantive faith-sensitive culturally competent DVA sector, it is less clear to what degree this is the case for countries outside of Euro-America. Studies from religious societies in Africa, Asia and the Levant suggest that religious leaders are oftentimes at the forefront of responding to family issues, including DVA, and may comprise an informal support sector (Istratii, 2020; Le Roux and Pertek, 2023). In the international development sector, it is well-established that faith-based organizations often engage in faith-informed ways to respond to DVA (Istratii and Kalkum, 2023). Such approaches, perhaps because they are underrepresented in the Anglo-American academic literature or considered too different from organized DVA services in industrialized societies have not been given sufficient attention, but could offer a wealth of experience for western industrialized societies to learn from.

Additionally, there were relatively few studies that robustly assessed the effectiveness of faith informed DVA services and limited evidence was found about the standardization and accreditation of faith sensitive DVA responses or services. As the evaluation by Stennis et al. (2015) suggests, a degree of standardization and patenting might be necessary for ensuring consistency in the delivery of faith-informed culturally competent or culturally appropriate programs. This might be an important matter to investigate further in order to establish how best to ensure that the specialization of culturally specific faith-informed services is recognized by and vis-à-vis generalist services to achieve their sustainability.

## Limitations

The review was limited by time and resources and did not include quality appraisal of primary studies. Additionally, while extensive searches were run to locate the most relevant studies and resources, it is very likely that relevant materials were missed due to: (a) related work being published under different fields of practice and terminologies, (b) organizational resources not surfacing in our online searches and (c) an inherent difficulty in defining organized DVA services, which this scoping review focussed on. We did not want to limit our search to high-income industrialized societies where organized DVA sectors are more visible or established, recognizing that many low- and middle-income societies cater to large refugee and migrant populations. Hence, we were open to including studies from around the world, however, many studies that emerged in the search referred to international development and public health interventions, typically externally funded programs led/introduced by external organizations. These were excluded in the current review as the focus was placed on organized country-led DVA services sectors; in addition, community-based responses working with faith, including those implemented in international development and public health contexts, had been reviewed in a previous paper (Istratii and Ali, 2023).

It is also significant that the review was limited to studies in English. It is reasonable to assume that many more relevant studies exist in national, regional or local languages that were simply not indexed and captured in our search and previous publications that we reviewed.

## Notes

1. IPV can be perpetrated by men or women in heterosexual or same sex relationships, though violence against women, particularly sexual violence is more prevalent, more severe, with worse health impact than IPV against men (see e.g., Office for National Statistics, 2016).

2. By DVA victims, we refer to individuals who are in a situation of victimization and by DVA survivors we refer to those who have managed to exit an abusive situation. The distinction aims to recognize the two different states, but it is understood that these can overlap. Both victims and survivors can be resilient and practice their agency to endure through, navigate, mitigate and exit their situation.
